# Kai-Xin-San Attenuates Doxorubicin-Induced Cognitive Impairment by Reducing Inflammation, Oxidative Stress, and Neural Degeneration in 4T1 Breast Cancer Mice

**DOI:** 10.1155/2021/5521739

**Published:** 2021-06-12

**Authors:** Wenjiao Lyu, Mingzi Ouyang, Xiaomeng Ma, Tiantian Han, Dajin Pi, Shijun Qiu

**Affiliations:** ^1^Department of Radiology, The First Affiliated Hospital of Guangzhou University of Chinese Medicine, Guangzhou, Guangdong 510405, China; ^2^Guangzhou University of Chinese Medicine, Guangzhou, Guangdong 510405, China; ^3^School of Traditional Chinese Medicine, Jinan University, Guangzhou, Guangdong 510632, China

## Abstract

**Objective:**

This study explored the potential therapeutic effect and possible mechanism of Kai-Xin-San (KXS) on doxorubicin-induced cognitive impairment in 4T1 breast cancer mice.

**Methods:**

A model of chemotherapy-induced cognitive impairment (CICI) was established with the injection of doxorubicin (DOX, 5 mg/kg) at a 7-day interval in a 4T1 breast cancer mouse. KXS was given (1 g/kg) daily by gavage over three weeks starting at the first week while giving DOX. The Morris water maze task was performed to measure the CICI-like behaviors. Oxidative stress markers in the hippocampus, inflammatory cytokines in the serum and hippocampus, Nissl staining, immunofluorescence staining, and analysis for Glial fibrillary acidic protein and ionized calcium-binding adapter molecule 1 of the hippocampus were examined to explore the effect and mechanism of KXS on DOX-induced CICI. Meanwhile, tumor growth and survival time were tested in this study.

**Results:**

CICI-like behaviors induced by DOX occurred earlier and were severer than the cognitive impairment induced by the tumor, and the effect of KXS on improving the cognitive impairment was obvious. KXS protected against DOX-induced neuroinflammation by decreasing levels of proinflammatory cytokines interleukin-6, interleukin-12p70, and tumor necrosis factor-alpha in both serum and brain and interleukin-1*β* in the brain, increasing the anti-inflammatory cytokines interleukin-4 in the serum and interleukin-10 in the hippocampus, and inhibiting the astrocytic hyperplasia and microglial polarization in the hippocampus. KXS reduced neural degeneration and protected against DOX-induced oxidative stress according to decreased malondialdehyde level, increased glutathione level, and enhanced activities of superoxide dismutase, catalase, and glutathione peroxidase. Moreover, KXS recovered the lost body weights after DOX administration and prolonged the survival times of mice.

**Conclusions:**

KXS may attenuate DOX-induced cognitive impairment by regulating inflammatory responses and reducing oxidative stress and neural degeneration. These findings also presented the role of KXS in improving the quality of life and prolonging survival time in breast cancer mice that received chemotherapy.

## 1. Introduction

Although great advancements in therapeutic methods have increased, chemotherapy is still the vital treatment for breast cancer [1]. Chemotherapy-induced cognitive impairment (CICI), one of the most serious adverse effects caused by chemotherapeutic treatments, has been observed in 69–78% of breast cancer patients [2], presenting as cognitive dysfunctions in memory, concentration, reasoning, and executive function during or following treatment [3], which affect the long-term quality of life or even result in the deaths of patients [4].

Previous studies show that the underlying mechanisms of CICI are connected with neural inflammatory response and oxidative stress especially in the brain [5–7]. Doxorubicin (DOX), a typical chemotherapeutic agent known for producing reactive oxygen species (ROS) and inducing oxidative stress in the tumor or even the whole body [5], is used commonly in adjuvant and neoadjuvant chemotherapy treatment for breast cancer [1]. Although DOX poorly crosses the blood-brain barrier (BBB), more and more experimental pieces of evidence show that DOX may cause many impaired cognitive functions [8, 9], which are also observed in clinical studies [10]. Furthermore, the overexpression of peripheral oxidative stress can increase the level of circulating tumor necrosis factor-alpha (TNF-*α*) to cross the BBB directly and activate the local generation of proinflammatory cytokines [11], such as interleukin-6 (IL-6), interleukin-1*β* (IL-1*β*), and TNF-*α* [12] as well as exacerbating oxidative stress in the brain [13]. The resulting neuroinflammation and oxidative stress can trigger apoptotic cell death and cause neurobehavioral alterations [7, 14]. Although these studies suggested that neuroinflammation, oxidative stress, and damages to neuronal structure and function might play a key role in DOX-induced cognitive dysfunction, definite and effective intervenes are still lacking.

Kai-Xin-San (KXS), a famous traditional Chinese medicine (TCM) prescription, composed of Ginseng Radix, Poria, Polygalae Radix, and Acori Tatarinowii Rhizoma (as shown in Supplementary Table 1), is widely used for the treatments of Alzheimer's disease (AD) [15], depression [16], and anxiety [17] clinically, alleviating symptoms as morbid forgetfulness, attention deficits, decreased executive functioning, and depression [18], which are very similar to the symptoms of CICI. Previous animal studies have proved that KXS improved cognitive disorders by influencing the inflammatory pathways [19–21], reducing apoptosis and oxidative stress [19, 22–24], protecting neurogenesis [25, 26], and regulating the expression of neurotrophic factor receptors [25] and synaptic proteins in the brain [27]. Based on the anti-neuroinflammation, antioxidative stress, and neuroprotective effects, we provided the hypothesis that KXS might have the effect of attenuating CICI, which has not been reported yet. In this study, we have focused on the spatial learning and working memory and the biochemical markers of KXS in the 4T1 murine breast cancer model treated with DOX to evaluate whether KXS has beneficial effects on CICI.

## 2. Materials and Methods

### 2.1. Materials and Reagents

DOX (10 mg) was bought from Hanhui Pharmaceutical Co., Ltd. (Hangzhou, China). All components of KXS were bought from Kangmei Pharmaceutical Co., Ltd. (Shenzhen, China). Reagent kits for the examination of cytokines (IL-1*β*, IL-6, TNF-*α*, IL-12p70, IL-4, and IL-10) and kits for the examination of oxidative stress markers malondialdehyde (MDA), glutathione (GSH), superoxide dismutase (SOD), catalase (CAT), and glutathione peroxidase (GPx) were obtained from Servicebio Institute of Biotechnology (Wuhan, China).

### 2.2. Preparation of KXS

All medicines formulating KXS were identified by Professor Shiyin Feng. Radix Ginseng (Ren shen), Poria (Fu ling), Radix Polygalae (Yuan zhi), and Rhizoma Acori Tatarinowii (Shi changpu) at a ratio of 3 : 3:2 : 2 were mixed and processed as reported previously [23]. 300 g Ginseng Radix, 300 g Poria, 200 g Polygalae Radix, and 200 g Acori Tatarinowii Rhizoma were soaked together in 10 L water for 3 h and extracted 2 times using a circumfluence extraction method. All extracts were combined and evaporated. Finally, the liquid after concentration was mixed to 0.1 g/mL and refrigerated at −20°C. Details of the herbal materials are listed in Supplementary Figure 1 and Supplementary Table 1.

The obtained KXS extracts were standardized using the high-performance liquid chromatography- (HPLC-) fingerprint method. Representative fingerprint chromatograms of KXS extracts are displayed in Supplementary Figure 2. By determining the chemical amounts in KXS, the contents of these chemicals should be no less than the values listed in Supplementary Table 2.

### 2.3. 4T1 Cell Culture

Murine breast carcinoma 4T1 cell strain was purchased from the Shanghai Institute of Biochemistry and Cell Biology of the Chinese Academy of Sciences (Shanghai, China). Cells were cultured by the RPMI 1640 medium including 10% inactivated fetal calf serum (Gibco, Brazil) and grown in an incubator with a humidified atmosphere of 5% CO_2_ and 37°C.

### 2.4. Animals

150 female BALB/c mice (16–20 g, 6–8 weeks old) were acclimatized for 7 days after being purchased from Medical Laboratory Animal Center of Guangdong province (Approval No. SCXK (Yue) 2018–0002, Foshan, China). Mice were housed 10/per cage, and the food and water were available ad libitum in the Animal Center of Guangzhou University of Chinese Medicine, accredited by IACUC (Institutional Animal Care and Use Committee). The room temperature was maintained at 20°C ± 1°C, and lights were kept from 8 : 00 am to 8 : 00 pm to imitate a 12 h light and 12 h dark cycle. The experiments were approved by the Animal Research Ethics Committee of Guangzhou University of Chinese Medicine (No. TCMF1-2019012), and after the study, the remaining mice were terminated individually and deeply anesthetized with pentobarbital. All applicable institutional and governmental regulations concerning the ethical use of animals were followed.

### 2.5. Experimental Design

**Figure 1:**
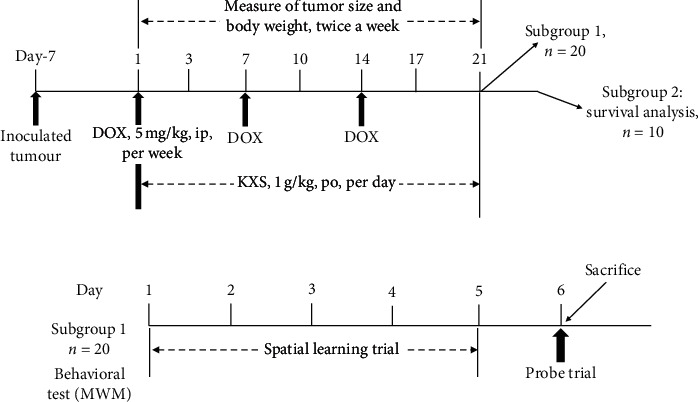
Experimental design and timeline of the study. (a) At the end of the duration of treatment, 20 mice were selected randomly from each group as subgroup 1 for behavioral test and the rest 10 mice as subgroup 2 for survival analysis. (b) MWM was taken to evaluate the cognitive impairment of mice; after the behavioral test, mice of subgroup 1 were sacrificed. DOX: doxorubicin; KXS: Kai-Xin-San; MWM: Morris water maze task.

150 mice were randomly divided into 5 groups. Except for the 30 mice of the NC group (normal control), the rest 120 mice were injected with a total of 1 × 10^5^ 4T1 cells in the right fourth mammary fat pad for establishing the tumor-bearing breast cancer model as the previous study described [28]. When the tumor was grown to a mean volume of 80–100 mm^3^ on day 7, mice were divided into 4 groups (*n* = 30): (1) TC group (tumor control), (2) TC + KXS group (tumor plus KXS treated), (3) DOX group (tumor plus DOX treated), and (4) DOX + KXS group (tumor plus DOX and KXS cotreated). Mice in the DOX and DOX + KXS groups were given DOX (5 mg/kg) weekly by intraperitoneal injection, and the dose and treatment duration of DOX were adopted based on previous study presenting the DOX-induced cognitive dysfunction and neurotoxicity [29]. Mice in the DOX + KXS and TC + KXS groups received KXS (1 g/kg, same as clinical equivalent dose) daily by gavage lasted for three weeks from the beginning of the first time DOX is given. The normal control and tumor control groups received injection and intragastric administration of normal saline (dose equaled to DOX and KXS). At the end of the duration of treatment, 20 mice were selected randomly from each group for behavioral tests (subgroup 1), and the rest 10 mice in each group were continued to feed for survival time recording (subgroup 2) (see Figure 1).

### 2.6. Behavioral Test

Morris water maze (MWM) task was taken to evaluate the spatial learning and working memory of mice. MWM task was measured in a circular pool filled with clean black water (50 cm height, 100 cm diameter, 20 ± 1°C) by finding a submerged platform. The platform (8 cm diameter) was transparent and placed 1 cm below the surface of water, where it was in the middle of the fourth quadrant of the pool. In order to help the mice to orientate and remember the platform, obvious and unaltered visual cues were placed on the walls around the pool, and if mice did not arrive at the platform within 60 s, experimenter would guide them to the platform for 10 s by a stick. All mice were required to test four times from four different entrances a day and lasted for five days. Then, 24 h after the last day of training, the platform was removed, and mice were given a 60 s retention probe test. Data were automatically collected by the Video Tracking System (Xinruan Biological Technology Co., Ltd, Shanghai, China).

### 2.7. Cytokines and Oxidative Stress Marker Measurement

24 h after the behavioral test, mice of each subgroup1 were anesthetized with isoflurane inhalation (RWD Life Science Pharmaceutical Co., Ltd., Shenzhen, China). When the mice were unconscious, the tumors were excised and weighed. The blood was collected via cardiac puncture and stored in 1.5 ml Eppendorf tubes for centrifuging at 3500 r/min for 7 min to separate the serum for cytokine measurement. Then, the brains of mice were immediately removed after cardiac perfusion with cold phosphate-buffered saline (PBS). The right hippocampus was dissected from the brains for the following analysis. The levels of cytokines, IL-1*β*, IL-6, TNF-*α*, IL-12p70, IL-4, and IL-10 in serum and hippocampus, the levels of MDA, GSH, and activities of SOD, CAT, and GPx in hippocampus were measured following the recommended procedures provided in the kit.

### 2.8. Immunohistological Examination

#### 2.8.1. Nissl Staining

The left hemispheres were removed from brains and fixed in 4% paraformaldehyde for 24 hours and then incubated in 30% sucrose for 48 hours. Hippocampal coronal sections 16 *μ*m thick were taken using a Thermo NH50 cryostat, and 0.5% crystal violet was used for Nissl staining as in the previous study [30]. The morphological changes of neurons in the different positions of the hippocampus were observed under a light microscope at 200x magnification by two independent observers.

#### 2.8.2. Immunofluorescence Staining and Analysis for GFAP and IBA-1

The rest sections were used for immunofluorescence staining for glial fibrillary acidic protein (GFAP, reactive astrocytes), ionized calcium-binding adapter molecule 1 (IBA-1, microglia), and 4′, 6-diamidino-2-phenylindole (DAPI, nuclei). After being blocked by 5% normal goat serum, 1% bovine serum albumin, and 0.5% TritonX in PBS, sections were incubated with rabbit anti-mouse GFAP (1 : 500; Servicebio) or rabbit anti-mouse IBA-1 antibody (1 : 2000; Servicebio) overnight at 4°C. The following day, sections were incubated with fluorochrome-conjugated secondary antibody (goat anti-rabbit, 1 : 200; Servicebio) for 1 hour and DAPI (0.01 mol/L in PBS, Servicebio) for 10 min successively at room temperature and then washed in PBS and mounted. Fluorescent images were captured on a NIKON Eclipse ci microscope. 5-6 representative images of each mouse were taken at 400x magnification in the hippocampus and analyzed by blinded investigators with ImageJ.

#### 2.8.3. Examination of the Growth of Tumor, Body Weight, and Survival Time

The size of the tumor was measured twice a week by a caliper and calculated with the formula as previously described [28]. And the body weight of mice was weighed at the same frequency. All these surveys lasted 3 weeks; then, 10 mice were selected randomly from each group for survival analysis in the following 30 days. After the study, the remaining mice were terminated individually and deeply anesthetized with pentobarbital sodium (100 mg/kg, ip, Sigma Chemical Company, MO, USA).

#### 2.8.4. Statistical Analysis

SPSS 22.0 software was used for statistical analysis. Examination of tumor volume, body weight, and escape latency was determined by repeated analysis of variance (ANOVA). Other behavioral tests, cytokines, oxidative stress markers, and immunohistological examination were detected by one-way ANOVA. Survival time analysis was examined using Kaplan-Meier. Data were expressed as mean ± standard error of the mean (SEM). A difference of *P* < 0.05 was considered statistically significant.

## 3. Results

### 3.1. Effects on Cognitive Impairment Behaviors

**Figure 2:**
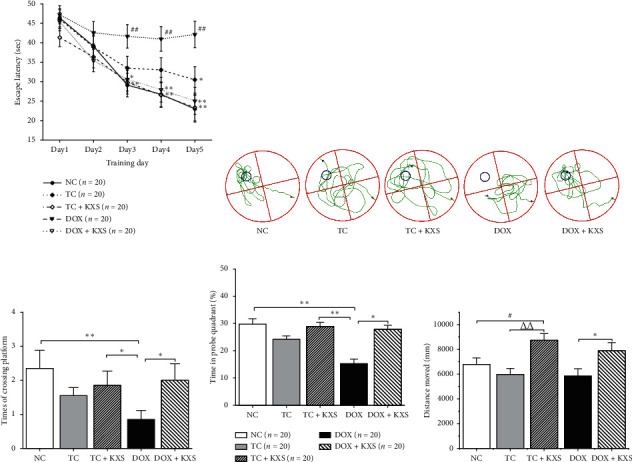
Effects on Morris water maze task. 5-day training trials: escape latency to a hidden platform (a), probe test: swimming path (b), times of crossing platform location (c), time in probe quadrant (d), and distance moved (e). *n* = 20, ^#^*P* < 0.05, ^##^*P* < 0.01, and ^###^*P* < 0.001 compared to the NC group; ^△^*P* < 0.05, ^△△^*P* < 0.01, and ^△△△^*P* < 0.001 compared to the TC group; ^*∗*^*P* < 0.05, ^*∗∗*^*P* < 0.01, and ^*∗∗∗*^*P* < 0.001 compared to the DOX group.

MWM task was taken to determine the spatial learning and working memory of mice. The escape latency to the platform in a 5-day training trial was assessed for spatial learning. Repeated measures ANOVA revealed significant effects of groups (*F*_4,16_ = 7.453, *P* < 0.001) and time (*F*_4,16_ = 26.18, *P* < 0.001) on the escape latency. Over time, the effect of repeated learning had differential effects on the groups. The DOX group showed a longer time to arrive at the platform compared with the NC group from day 3 to day 5 (*P* < 0.001) and the TC group on day 5 (*P* < 0.05). The DOX + KXS group remarkably shorted the escape latency at days 3–5 in comparison with the DOX group (*P* < 0.001), and the latency of the TC + KXS group was shorter than the TC group at day 5 (*P* < 0.05), consistent with the NC group (see Figure 2(a)).

The probe test was conducted approximately 24 h after the last training trial. In the test, the crossing times on the location where the platform used to locate were recorded to evaluate spatial memory. Group effects on crossing times (*F*_4,95_ = 3.288, *P* < 0.05) (see Figure 2(c)), duration spent in the targeted quadrant (*F*_4,95_ = 2.947, *P* < 0.05) (see Figure 2(d)), and distance moved (*F*_4,95_ = 4.930, *P* < 0.05) (see Figure 2(e)) were observed to be significantly different. The crossing times and duration on the targeted location of the DOX group were markedly less than the NC group, but the two variables were significantly greater in DOX + KXS group than in the DOX group (*P* < 0.05). It was also found that the mice of TC + KXS and DOX + KXS groups moved more distance than TC and DOX groups (*P* < 0.05), but there was no difference among the NC, TC, and DOX groups in the moved distance.

### 3.2. Effects on Volume and Weight of Tumor

**Figure 3:**
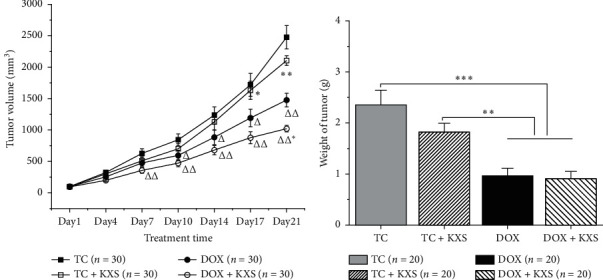
Effects on volume and weight of the tumor. Tumor volume of the 4T1 inoculated breast cancer model (a), mean ± SEM: *n* = 30, ^△^*P* < 0.05, ^△△^*P* < 0.01, and ^△△△^*P* < 0.001 compared to the TC group; ^*∗*^*P* < 0.05, ^*∗∗*^*P* < 0.01, and ^*∗∗∗*^*P* < 0.001 compared to the DOX group. Tumor weight of the 4T1 inoculated breast cancer model (b), mean ± SEM: *n* = 20, ^*∗*^*P* < 0.05, ^*∗∗*^*P* < 0.01, and ^*∗∗∗*^*P* < 0.001 compared to DOX and DOX + KXS groups.

The antitumor effects of KXS on a 4T1 inoculated breast cancer model independently and cotreated with DOX were assessed by measuring the volume and weight of the tumor. Significant effects of the interaction between days and groups were observed (days, *F*_6,124_ = 143.68, *P* < 0.001; day ∗ group, *F*_18,378_ = 4.154, *P* < 0.001) on the tumor's volume. As shown in Figure 3(a), treatments with DOX + KXS and DOX decreased the rates of tumor growth starting on day 7 and day 10, respectively, compared with the TC group (*P* < 0.05). DOX + KXS group showed a declining trend of tumor growth at day 21 (*P* < 0.05) but no significant difference in tumor weight in comparison with the DOX group. And there was no obvious difference between TC + KXS and TC groups. The tumor inhibition rates of TC + KXS, DOX, and DOX + KXS were 22.6%, 58.8%, and 59.3% (compared with the TC group, *P*=0.06, 0.001, and 0.001, respectively) (see Figure 3(b)).

### 3.3. Effects on Body Weight and Survival Time

**Figure 4:**
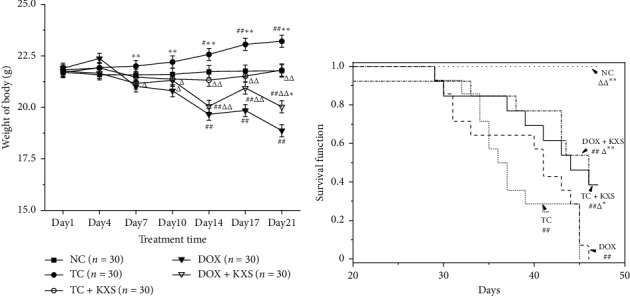
Effects on body weight and survival time. Body weight of mice (a), mean ± SEM: *n* = 30; survival time of mice (b), *n* = 10. ^#^*P* < 0.05, ^##^*P* < 0.01, and ^###^*P* < 0.001 compared to the NC group; ^△^*P* < 0.05, ^△△^*P* < 0.01, and ^△△△^*P* < 0.001 compared to the TC group; ^*∗*^*P* < 0.05, ^*∗∗*^*P* < 0.01, and ^*∗∗∗*^*P* < 0.001 compared to the DOX group.

The body weight of mice was measured at regular intervals too. During the treatment period, there were significant effects of the interaction between days and groups (days, *F*_6,150_ = 16.457, *P* < 0.001; day ∗ group, *F*_24,630_ = 8.591, *P* < 0.001). It was found that the body weight of the TC group was obviously increasing from day 14 compared to NC and TC + KXS groups (*P* < 0.05), where the two groups did not have a prominent difference. Moreover, mice of DOX and DOX + KXS groups lost body weight after every DOX injection and failed to return to the baseline body weight after the last treatment. However, the body weight of DOX + KXS group was heavier compared to the DOX group (*P* < 0.05) (see Figure 4(a)).

The rest 10 mice of each subgroup 2 were observed for survival analysis. By the end of the experiment, all the mice of the NC group were still alive, while the other four groups presented declining survival time (log Rank, *P* < 0.001). The survival time in the TC + KXS and DOX + KXS groups was significantly prolonged compared to the TC group (*P* < 0.05) and DOX group (*P* < 0.05) (see Figure 4(b)).

### 3.4. Effects on Serum and Hippocampus Cytokine Levels

**Figure 5:**
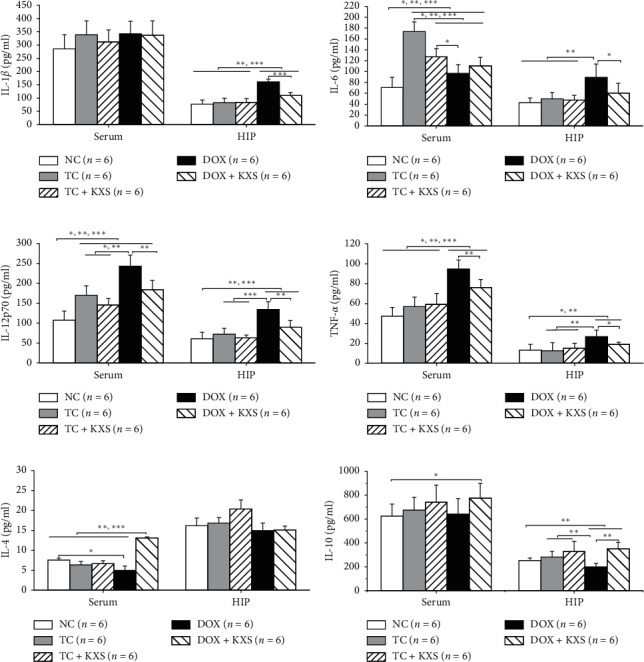
Effects on cytokine levels of serum and hippocampus. IL-1*β*, interleukin-one beta (a); IL-6, interleukin-six (b); IL-12p70, interleukin-12 protein 70 (c); TNF-*α*, tumor necrosis factor-alpha (d); IL-4, interleukin-four (e); IL-10, interleukin-10 (f). Mean ± SEM, *n* = 6. ^*∗*^*P* < 0.05, ^*∗∗*^*P* < 0.01, and ^*∗∗∗*^*P* < 0.001 compared to each group.

IL-1*β*, IL-6, TNF-*α*, IL-12p70, IL-4, and IL-10 levels were examined in serum and hippocampus. Significant group effects were presented on IL-6, IL-12p70, and TNF-*α* levels in the serum and hippocampus (*F*_4,25_ ≥ 6.215, *P* < 0.001), IL-1 and IL-10 levels in the hippocampus (*F*_4,25_ ≥ 7.741, *P* < 0.001), and IL-4 level in serum (*F*_4,25_ = 12.880, *P* < 0.001). As shown in Figures 5(a)–5(f), DOX induced an obvious increase in the levels of IL-6, IL-12p70, and TNF-*α* in both serum and hippocampus and IL-1*β* in the hippocampus compared to the NC group (*P* < 0.001). Cotreatment with KXS decreased the levels of IL-12p70 and TNF-*α* in serum (*P* < 0.05) and IL-1*β*, IL-6, IL-12p70, and TNF-*α* in hippocampus upregulated by DOX. The serum levels of IL-6 and IL-12p70 in TC and TC + KXS groups were also higher than those of the NC group (*P* < 0.05), but TC + KXS group presented a lower serum level of IL-6 compared to the TC group (*P* < 0.05). The levels of IL-4 in serum and IL-10 in the hippocampus were decreased in the DOX group (*P* < 0.001), and these declined cytokine levels were significantly elevated by KXS supplementation (*P* < 0.001).

### 3.5. Effects on GFAP and IBA-1 Immunoreactivities

**Figure 6:**
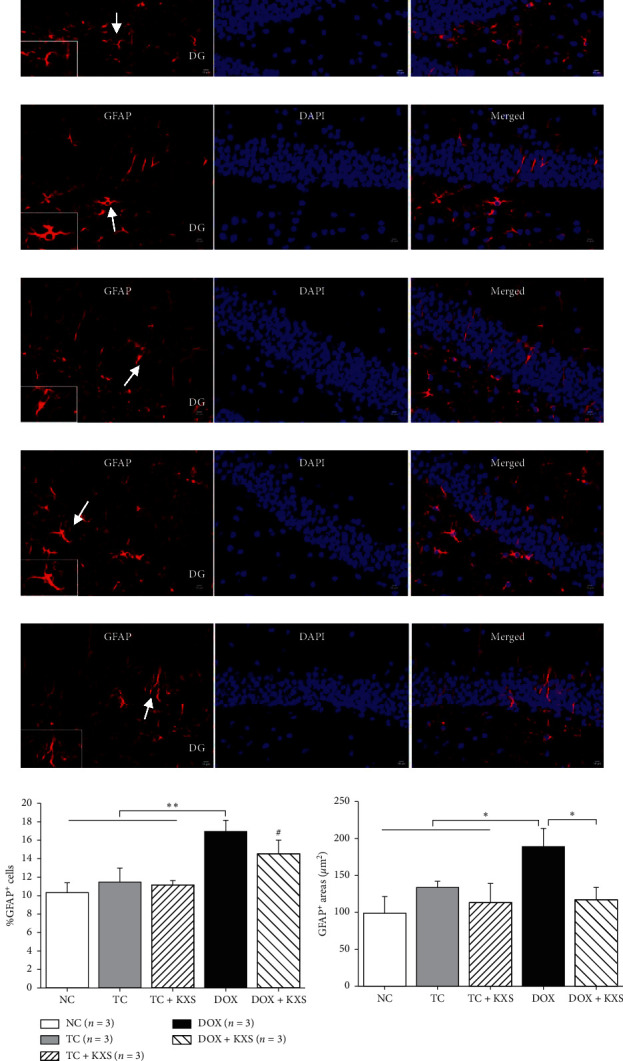
GFAP immunostaining in the mice hippocampus. Merged overview of the relevant hippocampus region with GFAP in red and nuclear counterstaining with DAPI in blue (magnification 400x). The white arrow depicts the GFAP^+^ cell shown in the enlarged image. NC group: very mild level of GFAP (a); TC group: moderate level of GFAP (b); TC + KXS group: very mild level of GFAP (c); DOX group: severe level of GFAP (d); DOX + KXS group: moderate level of GFAP (e). IH, scale bar = 50 *μ*m, *n* = 3/per group. GFAP: Glial fibrillary acidic protein; DAPI: 4′,6-diamidino-2-phenylindole; DG: dentate gyrus. The GFAP^+^ cells (f) (i) and the area of GFAP^+^ in the DG (f) (ii). Mean ± SEM, ^*∗*^*P* < 0.05, ^*∗∗*^*P* < 0.01, and ^*∗∗∗*^*P* < 0.001 compared to the DOX group, ^#^*P* < 0.05, ^##^*P* < 0.01, and ^###^*P* < 0.001 compared to the NC group.

**Figure 7:**
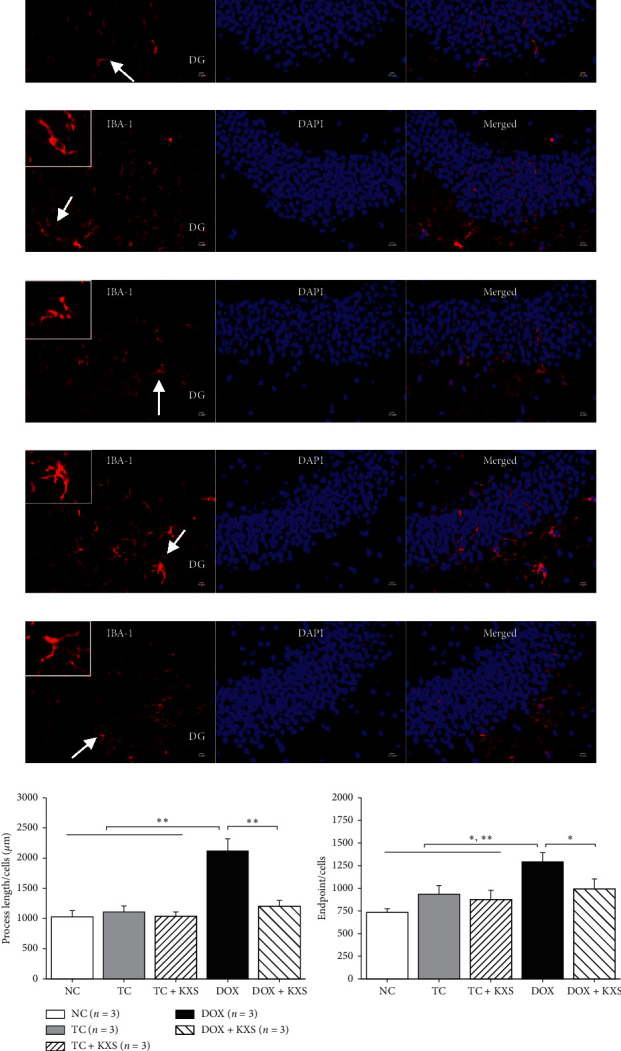
IBA-1 immunostaining in the mice hippocampus. Merged overview of the relevant hippocampus region with IBA-1 in red and nuclear counterstaining with DAPI in blue (magnification 400x). The white arrow depicts the IBA-1^+^ cell shown in the enlarged image. NC group: very mild level of IBA-1 (a); TC group: moderate level of IBA-1 (b); TC + KXS group: very mild level of IBA-1 (c); DOX group: severe level of IBA-1 (d); DOX + KXS group: IBA-1 expressions at a moderate level of IBA-1 (e). IH, scale bar = 50 *μ*m, *n* = 3/per group. IBA-1: ionized calcium-binding adapter molecule; DAPI: 4′,6-diamidino-2-phenylindole; DG: dentate gyrus. The process length of IBA-1^+^ cells (f) (i), the endpoint of IBA-1^+^ cells in the DG (f) (ii). mean ± SEM, ^*∗*^*P* < 0.05, ^*∗∗*^*P* < 0.01, and ^*∗∗∗*^*P* < 0.001 compared to the DOX group.

To investigate the neuroinflammation of mice brains, GFAP labeling was used to examine activated astrocytes, and IBA-1 labeling was used to examine microglia. As shown in Figures 6(a)–6(e), GFAP immunoreactivity was increased in DOX and DOX + KXS groups in the dentate gyrus (DG) of the hippocampus. The analysis of the threshold images indicated that there were significant group effects on the astrocytic cell number (*F*_4,10_ = 15.782, *P* < 0.0001) and astrocytic area (*F*_4,10_ = 8.724, *P* < 0.01). DOX group presented increased GFAP^+^ cell number and area compared to NC, TC, and TC + KXS groups (*P* < 0.01). The astrocyte area of the DOX + KXS group was remarkably reduced (*P* < 0.01) although the GFAP^+^ cell number did not present a significant difference compared with the DOX group (see Figure 6(f)). As shown in Figures 7(a)–7(e), the DOX group presented obviously increasing IBA-1 immunoreactivity in DG. The analysis of the threshold images indicated that there were significant group effects on the microglial cell endpoint (*F*_4,10_ = 9.734, *P* < 0.01) and microglial process length (*F*_4,10_ = 29.144, *P* < 0.0001). It was found that the IBA-1^+^ cell endpoint and process length of the groups treated with DOX were increased significantly compared to the NC group (*P* < 0.01), while the tumor did not have a remark effect on the immunoreactivity in the brain. However, the KXS + DOX group significantly reduced the IBA-1^+^ cell endpoint and process length compared with the DOX group (*P* < 0.01) (see Figure 7(f)).

### 3.6. Effects on the Hippocampus MDA, GSH Levels, and Antioxidant Enzyme Activities

**Figure 8:**
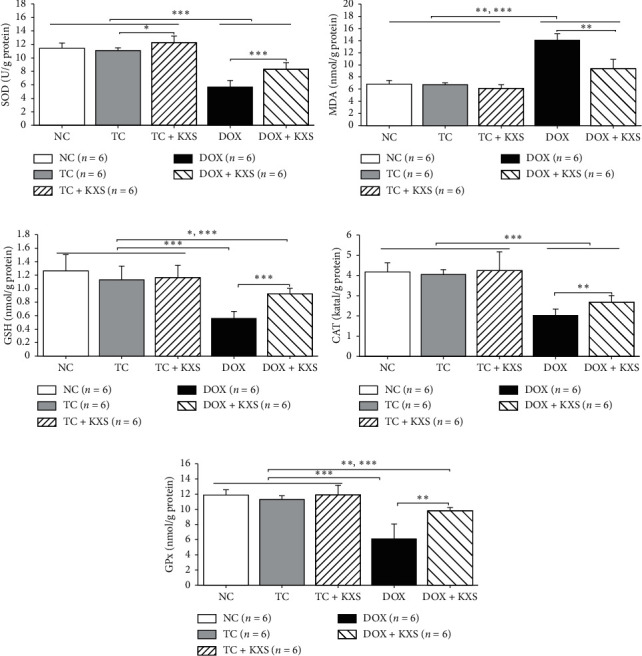
Effects on the hippocampus MDA, GSH level, and antioxidant enzyme activities. SOD, superoxide dismutase (a); MDA, malondialdehyde (b); GSH, glutathione (c); CAT, catalase (d); GPx, glutathione peroxidase (e). mean ± SEM, *n* = 6. ^*∗*^*P* < 0.05, ^*∗∗*^*P* < 0.01, and ^*∗∗∗*^*P* < 0.001 compared to each group.

There were significant group effects on the hippocampus MDA, GSH levels, and antioxidant enzyme activities (SOD, GPx, and CAT) (*F*_4,25_ ≥ 15.955, *P* < 0.0001). In this study, the MDA level in hippocampus was increased markedly in DOX-treated groups (*P* < 0.01) and unchanged in the TC and TC + KXS groups compared to the NC group (*P* > 0.05). Meanwhile, KXS + DOX group presented lower level of MDA compared to the DOX group (*P* < 0.01) (see Figure 8(b)). In addition, the level of GSH and the activities of SOD, GPx, and CAT in hippocampus of the groups treated with DOX were significantly lower than those of the NC group (*P* < 0.01), and the tumor did not have remark effect on these enzyme activities. Nevertheless, KXS cotreatment significantly increased the activities of SOD, GPx, and CAT and improved the GSH level compared with the DOX group (*P* < 0.01). It was also demonstrated that the SOD activity in the TC + KXS group was higher than that in the TC group (*P* < 0.05) (see Figures 8(a) and 8(c)–8(e)).

### 3.7. Effects on Neural Degeneration

**Figure 9:**
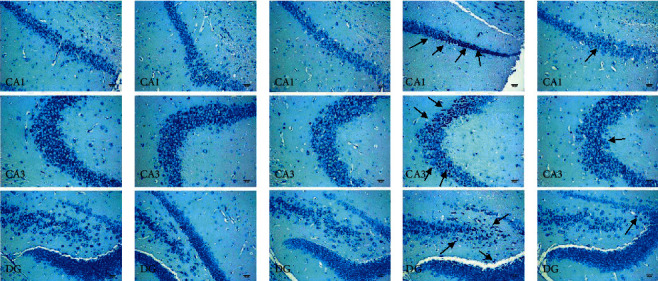
Effects on Nissl staining results of the hippocampus. The black arrow depicts the degenerative changes of the neurons in the CA1, CA3, and DG sectors. NC group (a); TC group (b); TC + KXS group (c); DOX group, presented severe neural degeneration signs as shrunken cytoplasm and condensed (d); DOX + KXS group, presented moderate amount of degenerative neurons (e). Scale bar = 50 *μ*m, *n* = 3/per group, DG, dentate gyrus.

In this study, Nissl staining of the hippocampus was detected. As shown in Figures 9(a)–9(e), the DOX group showed severe degenerative changes of neurons as the morphology of shrunken cytoplasm and condensed staining compared with NC, TC, and TC + KXS groups in the CA1, CA3, and DG sectors of the hippocampus, whereas those neural degeneration signs were alleviated in the DOX + KXS group.

## 4. Discussion

As a well-established and highly effective chemotherapeutic drug, the use of DOX especially in the treatment of breast cancer tends to induce neurotoxicity and lead to CICI. In the current study, it was found that KXS reduced learning and memory impairments and inhibited inflammation, oxidative stress, and neural degeneration in a 4T1 breast cancer mouse model of DOX-induced CICI.

MWM task was taken to evaluate the cognitive impairment of mice. DOX group demonstrated obvious impaired spatial learning and working memory abilities manifested as longer escape latency to the platform and fewer crossing times into the target location. Meanwhile, the TC group exhibited an increasing trend and showed significantly longer escape latency until day 5 compared with the NC and TC + KXS groups, but there were no significant effects on duration and crossing times. The total distance traveled of the NC, TC, and DOX groups in the task did not present a significant difference; however, the two groups treated with KXS (TC + KXS, DOX + KXS) showed more activities for their more distance moving. Previous animal studies about CICI have been conducted on normal rodents [31]. In our studies, CICI induced by DOX in a 4T1 breast cancer cell inoculated mouse model which has similarities to the chemotherapeutic process which occurs in human. These results suggested that the impairments of spatial learning ability and working memory caused by DOX occurred earlier and severer compared with the cognitive impairment induced by the presence of tumor, which was consistent with previous studies where some cancer patients were observed to have severer cognitive dysfunctions after receiving chemotherapy [10, 32, 33]. In addition, the effect of KXS on improving cognitive impairments was obvious.

Common cancer treatments of breast cancer, especially chemotherapy, could cause the overproduction of inflammatory cytokines, which may be correlated with CICI [12, 14]. Consequent neuroinflammation induced by peripheral inflammatory plays a critical role in the pathogenesis of cognitive dysfunctions [34, 35]. In this study, levels of proinflammatory cytokines (IL-1*β*, IL-6, IL-12p70, and TNF-*α*) and anti-inflammatory cytokines (IL-4 and IL-10) in the serum and hippocampus of mice were measured. In the current study, it was found that DOX not only increased the levels of IL-6, IL-12p70, and TNF-*α* in the serum and hippocampus and IL-1*β* in the hippocampus but also reduced the levels of IL-4 in the serum and IL-10 in the hippocampus. KXS widely restrained the increase of the proinflammatory cytokines and effectively recovered the levels of the anti-inflammatory cytokines induced by DOX. IL-6 level in the serum of the TC group was also increased, although there was not any significant effect on the hippocampus. In the brain, microglia and astrocytes are the important source of proinflammatory cytokines and play dynamic roles in coordinating responses between the immune system and brain [36, 37]. It has been proved that persistent glial reactivity may produce exaggerated levels of proinflammatory cytokines resulting in impaired cognitive performance [38, 39]. In this study, DOX increased the hyperplasia and overexpression of GFAP^+^ and IBA-1^+^ cells in hippocampus tissues, which suggested that DOX promoted the activation of astrocytes and the polarization of microglia; as expected, KXS cotreatment reversed this process. The above results suggested that DOX induced the dysregulation of inflammatory response in periphery and brain, and tumor on their own exhibited increased tendency of proinflammatory cytokines in periphery but not in the brain, which was consistent with previous clinical studies presenting that similar dysregulation of circulating cytokines was observed during the treatment of breast cancer [14, 40]. It also provided further evidence that KXS protected against DOX-induced neuroinflammation by decreasing levels of proinflammatory cytokines in both serum and brain and glial reactivity in the hippocampus and increasing the anti-inflammatory cytokines in both serum and brain.

**Figure 10:**
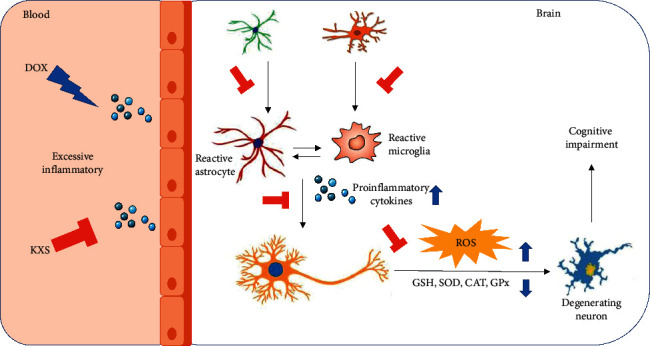
Potential mechanisms of the effects of KXS against CICI induced by DOX.

Previous studies showed that oxidative stress in the brain was correlated with neuroinflammation and neural degeneration, which led to memory-related neurodegenerative disorders [5]. In the process of oxidative stress, a decrease in GSH level [41] and an excessive amount of ROS can ultimately produce MDA [42]. And the removal of these productions of oxidative stress depends on the activity of antioxidant defensive systems including SOD, CAT, and GPx [43]. There has been increasing evidence that KXS has beneficial activities as antioxidants by increasing the activity or expression of endogenous antioxidant enzymes and reducing the apoptosis of neurons in the brain [20, 23, 44]. In this study, the degree of oxidative stress in the hippocampus was determined by measuring the levels of MDA and GSH and the activities of SOD, CAT, and GPx enzymes. It was seen that the DOX group increased the MDA level and decreased the GSH level and SOD, CAT, and GPx enzyme activities significantly in the hippocampus; however, these phenomena were reversed at significant levels in the DOX + KXS group. It was also shown that the SOD activity of the TC + KXS group was higher than that of the TC group while the tumor did not have any effects on these enzyme activities. Hippocampal neurons were also observed under the microscope, and it was found that DOX induced severe neural damage and degeneration in the hippocampus, whereas KXS cotreatment attenuated these injuries. These results suggest that KXS may reduce neural degeneration and alleviate DOX-induced oxidative stress according to decreased MDA level, increased GSH level, and enhanced antioxidant enzyme activities. Previous research proved that KXS modulated the aberrant upregulation of ROS in AD rats. Another study presented that KXS enhanced the SOD activity and decreased the MDA level in the serum of the fatigued rats [22]. These studies support the results obtained from our study (see Figure 10).

In our study, CICI was induced by DOX in a 4T1 murine breast cancer model, and the antitumor effect and toxicity of KXS were determined simultaneously. It was found that KXS could recover the lost body weight after DOX administration and prolong the survival time of mice although there were no significant antitumor effects on KXS-treated groups. These results indicated that KXS might be beneficial to improve the quality of life of DOX-treated tumor-bearing mice.

## 5. Conclusions

In conclusion, current results in this study suggested that KXS may attenuate DOX-induced cognitive impairment by regulating inflammatory responses and reducing oxidative stress and neural degeneration; these findings also presented the role of KXS in improving quality of life and prolonging survival time in breast cancer mice that received chemotherapy.

## Data Availability

The datasets used and/or analyzed during the current study are available from the corresponding author on reasonable request.

## Abbreviations

CICI: Chemotherapy-induced cognitive impairment
DOX: Doxorubicin
BBB: Blood-brain barrier
TNF-*α*: Tumor necrosis factor-*α*
IL-6: Interleukin-6
IL-1*β*: Interleukin-1*β*
IL-12p70: Interleukin-12p70
IL-4: Interleukin-4
IL-10: Interleukin-10
MDA: Malondialdehyde
SOD: Superoxide dismutase
GSH: Glutathione
CAT: Catalase
GPx: Glutathione peroxidase
MWM: Morris water maze task
GFAP: Glial fibrillary acidic protein
IBA-1: Ionized calcium-binding adapter molecule 1
DAPI: 4′,6-diamidino-2-phenylindole
DG: Dentate gyrus
NC group: Normal control group
TC group: Tumor control group
TC + KXS group: Tumor plus Kai-Xin-San-treated group
DOX group: Tumor plus doxorubicin-treated group
DOX + KXS group: Tumor plus doxorubicin and Kai-Xin-San-cotreated group.

## Supplementary Materials

Supplementary Materials The pictures and descriptions of the plants used by KXS, as well as the representative fingerprint chromatograms of the main active biochemical components of KXS decoction.
