# Supplementary material for: Kai-Xin-San Attenuates Doxorubicin-Induced Cognitive Impairment by Reducing Inflammation, Oxidative Stress, and Neural Degeneration in 4T1 Breast Cancer Mice
Source: Evid Based Complement Alternat Med. 2021 Jun 12;2021:5521739. doi: 10.1155/2021/5521739 (PMC8216823; doi:10.1155/2021/5521739)
Supplement: Supplementary Materials — The pictures and descriptions of the plants used by KXS, as well as the representative fingerprint chromatograms of the main active biochemical components of KXS decoction. [file 5521739.f1.docx]

### Materials and reagents

### Preparation of KXS

All medicines formulating KXS were identified by Professor Shiyin Feng. Ginseng Radix (Ren shen), Poria (Fu ling), Polygalae Radix (Yuan zhi) and Acori Tatarinowii Rhizoma (Shi changpu) at a ratio of 3:3:2:2 were mixed and processed as reported previously. [23] 300g Ginseng Radix, 300g Poria, 200g Polygalae Radix and 200g Acori Tatarinowii Rhizoma were soaked together in 10 L water for 3h and extracted 2 times using a circumfluence extraction method. All extracts were combined and evaporated. Finally, the liquid after concentrated were mixed to 0.1g/mL and refrigerated at -20℃. Details of the herbal materials are listed in Supplementary Figure 1 and Table 1.


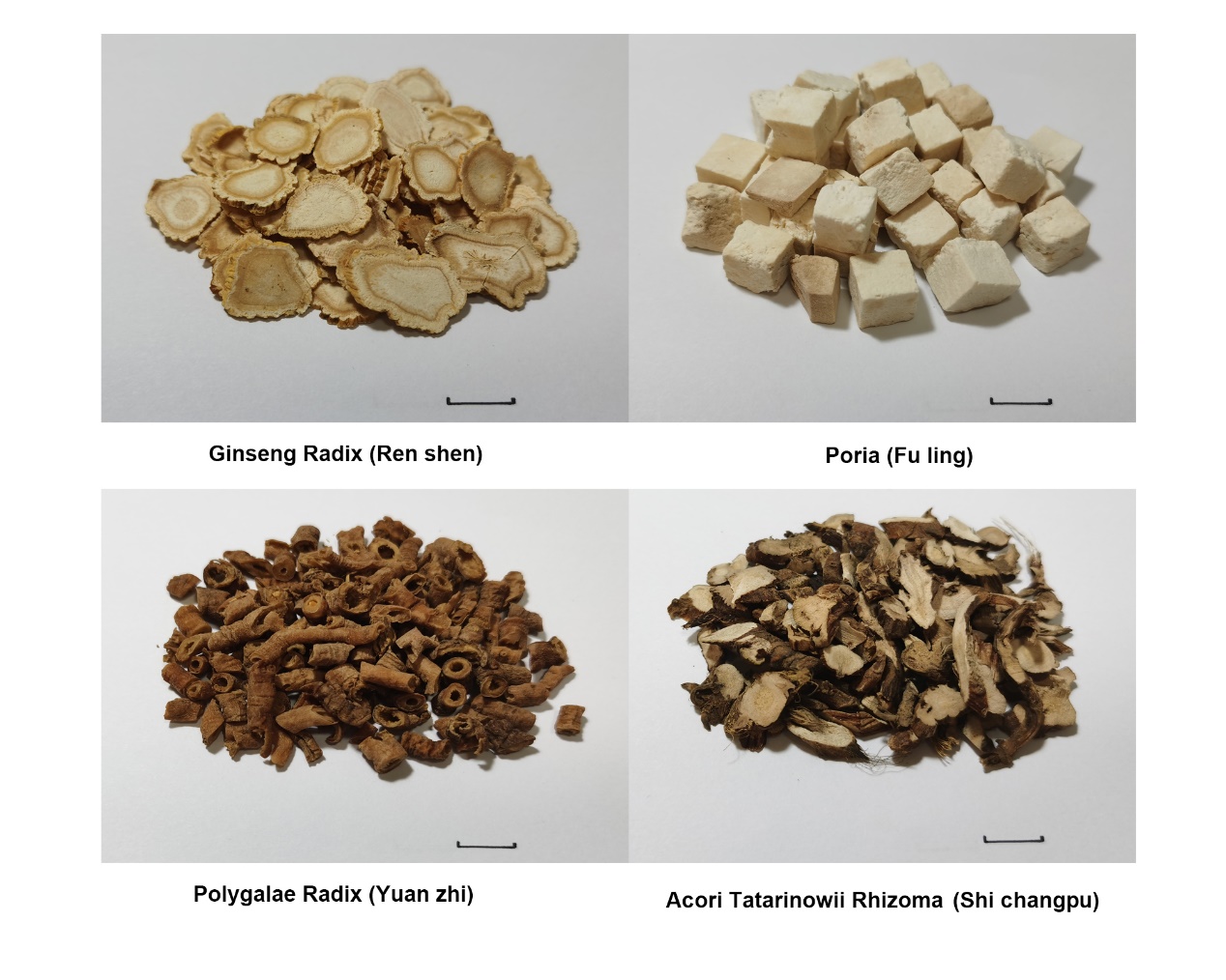


Supplementary Figure 1: Representative figures of herbs in KXS. Bar = 1 cm.

Supplementary Table 1: List of botanical, herbal, Chinese name and the producing location of the corresponding herb in KXS with its voucher number.

| **Biotanical name** | **Herbal name** | **Chinese name** | **Producing location** | **Voucher NO.** |
| --- | --- | --- | --- | --- |
| *Panax ginseng* C.A.Mey. | Ginseng Radix et Rhizoma | Ren Shen | Jilin, China | KGZ201901 |
| *Poria cocos* (Schw.) Wolf. | Poria | Fu Ling | Hunan, China | KGZ201902 |
| *Polygala tenuifolia* Wild. | Polygalae Radix | Yuan Zhi | Shanxi, China | KGZ201903 |
| *Acorus tatarinowii* Schott. | Acori Tatarinowii Rhizoma | Shi Chang Pu | Sichuan, China | KGZ201904 |

The obtained KXS extracts were standardized using a high-performance liquid chromatography (HPLC)-fingerprint method. Representative fingerprint chromatograms of KXS extracts are displayed in Supplementary Fig. 2. By determining the chemical amounts in KXS, the contents of these chemicals should be no less than the values listed in Supplementary Table 2.




Supplementary Figure 2: Chemical fingerprint chromatograms of KXS. Fingerprint chromatograms of KXS formulae were made by HPLC at a Welch Ultimate Plus-C18 column (250×4.6 mm, 5um), at a wavelength of 202 nm, a column temperature of 30°C, a flow rate of 1 mL/min, and an injection volume of 10μL. The identification of ginsenoside Rg1 (1), Rb1 (2), tenuigenin (3), β-asarone (4) and α-asarone (5) and pachymic acid (6) were shown in the chromatogram. The standard controls were purchased from Shanghai Yuanye Pharmaceutical Co., Ltd. (Shanghai, China)**.**

Supplementary Table 2: Criteria for standardized KXS

| **Herbal name** | **Chemical marker** | **Value** |
| --- | --- | --- |
| Ginseng Radix et Rhizoma | Ginsenoside Rg1 | 1311.2±15.8 |
|  | Ginsenoside Rb1 | 1539.9±39.8 |
| Poria | Pachymic acid | 0.4±0.1 |
| Polygalae Radix | Tenuigenin | 183.0±5.6 |
| Acori Tatarinowii Rhizoma | β-Asarone | 384.9±15.6 |
|  | α-Asarone | 6.8±0.6 |

Values were expressed in ug/100mg of KXS extracts, Mean ± SEM, n=3.
